# Family Presence during Resuscitation: A Qualitative Analysis from a National Multicenter Randomized Clinical Trial

**DOI:** 10.1371/journal.pone.0156100

**Published:** 2016-06-02

**Authors:** Carla De Stefano, Domitille Normand, Patricia Jabre, Elie Azoulay, Nancy Kentish-Barnes, Frederic Lapostolle, Thierry Baubet, Paul-Georges Reuter, Nicolas Javaud, Stephen W. Borron, Eric Vicaut, Frederic Adnet

**Affiliations:** 1 AP-HP, Urgences, Samu 93, hôpital Avicenne, 93000 Bobigny, France; 2 Paris 13 Sorbonne University, Paris Cité, EA 3509, 93000 Bobigny, France; 3 Inserm U970, Centre de Recherche Cardiovasculaire de Paris, Université Paris Descartes, Paris, France; 4 AP-HP, Samu de Paris, hôpital Necker-Enfants Malades, Paris, France; 5 AP-HP, réanimation médicale, hôpital Saint-Louis, Paris, France; 6 AP-HP, Department of Child and Adolescent Psychiatry and General Psychiatry, Avicenne Hospital, Paris, France; 7 Paris 13 Sorbonne University, Paris Cité, Laboratoire UTRPP (EA 4403), Inserm 669, France, 93000 Bobigny, France; 8 Department of Emergency Medicine, Texas Tech University HSC, El Paso, TX, United States of America; 9 AP-HP, Unité de Recherche Clinique, hôpital Fernand Widal, Paris, France; University of Oklahoma, UNITED STATES

## Abstract

**Background:**

The themes of qualitative assessments that characterize the experience of family members offered the choice of observing cardiopulmonary resuscitation (CPR) of a loved one have not been formally identified.

**Methods and Findings:**

In the context of a multicenter randomized clinical trial offering family members the choice of observing CPR of a patient with sudden cardiac arrest, a qualitative analysis, with a sequential explanatory design, was conducted. The aim of the study was to understand family members’ experience during CPR. All participants were interviewed by phone at home three months after cardiac arrest. Saturation was reached after analysis of 30 interviews of a randomly selected sample of 75 family members included in the trial. Four themes were identified: 1- choosing to be actively involved in the resuscitation; 2- communication between the relative and the emergency care team; 3- perception of the reality of the death, promoting acceptance of the loss; 4- experience and reactions of the relatives who did or did not witness the CPR, describing their feelings. Twelve sub-themes further defining these four themes were identified. Transferability of our findings should take into account the country-specific medical system.

**Conclusions:**

Family presence can help to ameliorate the pain of the death, through the feeling of having helped to support the patient during the passage from life to death and of having participated in this important moment. Our results showed the central role of communication between the family and the emergency care team in facilitating the acceptance of the reality of death.

## Introduction

The presence of the family during a patient’s cardiopulmonary resuscitation (CPR), successful or not, remains controversial.[1] Although increasingly recommended by various learned societies, health care personnel most often oppose this practice.[2–4] In a recent randomized clinical trial, we showed that family members systematically offered the choice of observing CPR had improved clinical indicators related to posttraumatic stress syndrome, better anxiety and depression scale scores, and less complicated grieving.[5,6] Moreover, the attendee’s presence did not increase the level of stress of the health care providers during the resuscitation.[5] Nonetheless, these relatives’ experience of both the choice to witness the CPR and of CPR itself has not yet been characterized.

An analysis of the literature finds some scattered themes potentially associated with the benefits or disadvantages of allowing families to be present during CPR.[1] These reports most often come from analyses of questionnaires or qualitative studies of family and close friends (summarized hereafter as family), professionals, or patients.[7–10] On the whole, very few of these studies have attempted to analyze systematically the themes identified in the recounted experiences of those present during a family member’s resuscitation. The themes reported include the need to see or touch the body, to be able to say a final goodbye, to be certain that everything that could be done was done.[1] Conversely, some authors have stressed the aggressive, traumatizing nature of the experience perceived by families present during CPR.[11]

The aim of this ancillary study of our clinical trial was to understand, through a systematic qualitative analysis, how families experience CPR of a relative, by detailing the emotional meaning of the benefits and disadvantages of their presence. This analysis viewed from the perspective of these family members, may help us to understand the results of the randomized trial, by showing the subjective factors that played a role and which may explain the difference found in the psychiatric morbidity between the group that was offered a choice and the group that was not.

## Methods

### Study context and Recruitment

This study, using a sequential explanatory design, is the qualitative component of a French randomized multicenter trial (the PRESENCE study) and explores the experiences of relatives related to their choice to be or not during CPR of a loved one. The decision to conduct a qualitative analysis was made at the outset, in the initial planning and methodological design. Briefly, the PRESENCE trial randomized 570 family members present in the homes of persons who had a sudden cardiac arrest and had CPR performed there by a team of paramedical and medical emergency care professionals. In the intervention group, the team routinely asked the family members if they wanted to be present during CPR. In the control group, there was no change from the usual management (which does not include that offer). The results for the intervention group showed a significant reduction in the rate of post-traumatic stress syndrome (PTSD), a reduction in anxiety and depression scores 90 days later,[5] and a favorable effect on the work of grieving at one year.[6]

**Fig 1:**
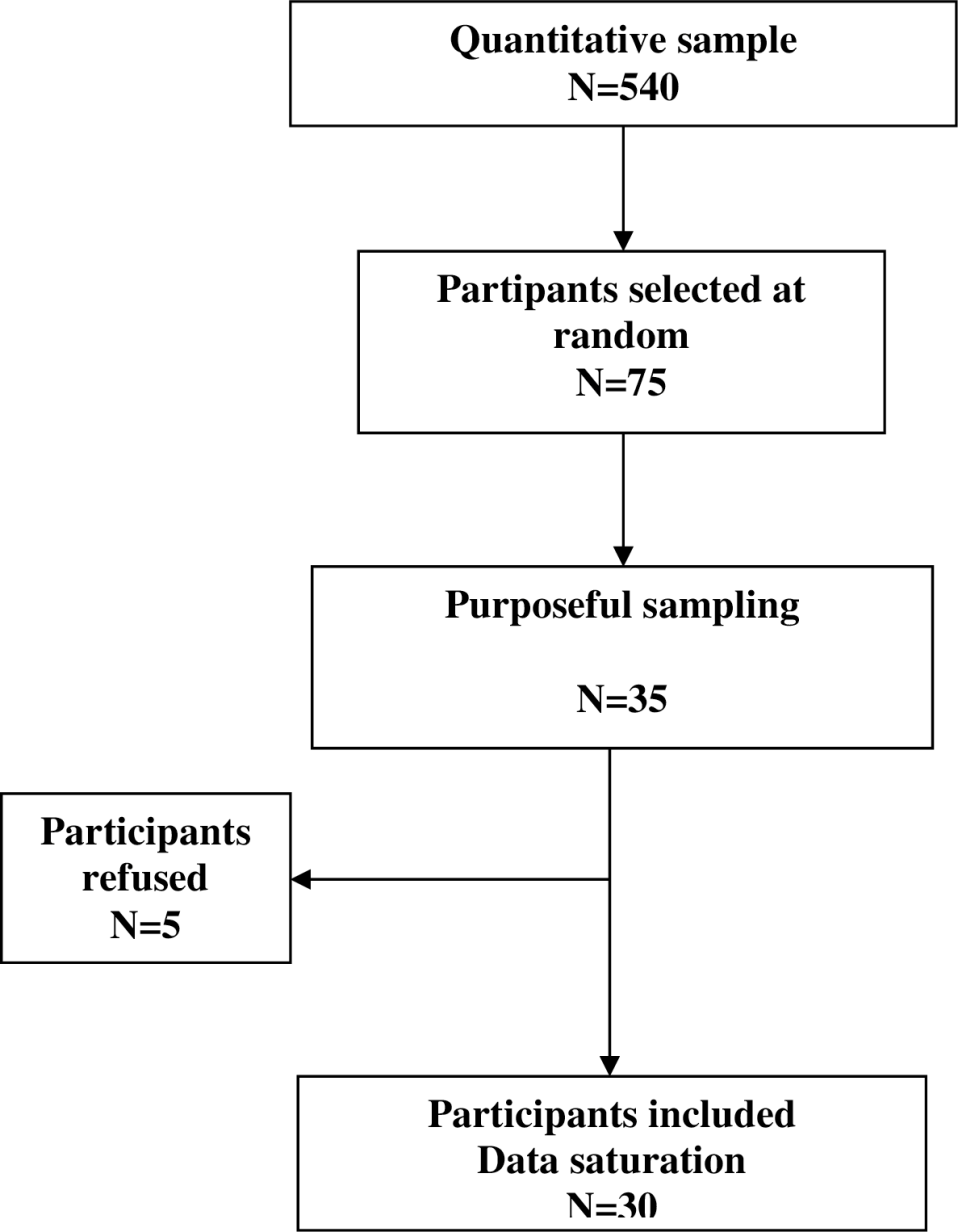
Flow chart of the sample.

Of the 540 participants included in the randomized controlled trial, 75 were randomly selected as possible candidates for the qualitative study. No family member of people who survived CPR was included. We contacted them sequentially by telephone according to an intentional sampling procedure known as purposive sampling (Fig 1). [12] This procedure was used to ensure exhaustiveness and to maximize the heterogeneity of the sample.

### Data collection

We interviewed family members of different age groups and different degrees of kinship, including both those who did and did not witness CPR. Of the first 35 subjects contacted, 5 refused to be interviewed and 30 were included in the analysis. This number was determined by data saturation.[13]

Three months after the patient's CPR at home, family members were contacted by a clinical psychologist (DN). The author (DM) who conducted the semi-structured interviews and the relatives interviewed no had direct experience or previous medical training/expertise in performing or witnessing resuscitations on acute cardiac patients. Each participant provided written consent, and the Patient Protection Committee of Aulnay-sous-Bois approved this study.

Semi-directive telephone interviews were conducted from June 2012 to October 2012, according to an interview guide drafted in advance (S1 Appendix). It explored 3 principal topics concerning the relative: (i) experience of the intervention by the emergency care team, whether or not the relative witnessed CPR; (ii) experience of presence at CPR; (iii) experience of not being present during CPR.

### Data Analysis

All interviews were recorded and transcribed verbatim. We applied a qualitative interpretative approach guided by grounded theory and based on a technique of constant comparison. There were three successive phases: open coding, axial coding, and selective coding. The analysis was performed independently by three researchers (CDS and DN) who met regularly during weekly meetings to discuss divergences during coding and to reach agreement (triangulation of the analysis).[13] NVivo software v. 10 (QSR International Ltd. 1999–2013) was used to facilitate the characterization of themes. This study is reported in accordance with the *COnsolidated criteria for REporting Qualitative* (COREQ) research statement.[14]

### Trial Registration

ClinicalTrials.gov number, NCT01009606.

### Institutional Review Board Approval

Ethics committee approval was given for this study by the Patient Protection Committee of Aulnays sous Bois. All participants gave informed consent before taking part and have given written consent to their interview data being included in publications.

## Results

Thirty interviews of family members were analyzed. Table 1 summarizes their demographic characteristics.

**Table 1:** Characteristics of the cohort.

Characteristics	Cohort study (N = 30)
Age, years—mean±SD	50±15
Male sex–no. (%)	9 (30)
Proposal to witness CPR–no. (%)	18 (60)
Relationship to patient–no. (%)	
Partner, husband or wife	17 (57)
Child	13 (43)
Parent	0 (0)
Sibling	0 (0)
Religion–no. (%) ^a^	
Catholic	14 (49)
Protestant	0 (0)
Jewish	0 (0)
Muslim	5 (17)
Other	0 (0)
No religion	10 (35)
Occupation, no. (%)	
Farmer	1 (3)
Employee, non managerial	11 (37)
Executive, manager	10 (33)
Professional	2 (7)
Unemployed	2 (7)
Retired	4 (13)

^a^1 missing data point

Four principal themes and 12 sub-themes were isolated (Tables 2 and 3).

**Table 2:** Themes and sub-themes of study.

Themes and sub-themes	No of participants	Percentage
Theme 1 –Choosing to be actively involved in the resuscitation	23	77%
Being present for CPR:		
To be actively involved in the resuscitation process	2	7%
To feel emotionally able to be present	3	10%
To support the patient during CPR	7	23%
To see the efforts of the resuscitation team	14	47%
Not being present for CPR:		
Wish to protect oneself	3	10%
Theme 2- Communication between the family member and the emergency team	27	90%
Medical information for the relative	14	47%
Satisfaction (or dissatisfaction) about the medical team’s intervention	5	17%
Theme 3- Perception of the reality of death	23	77%
Awareness of death at the arrival of the emergency team	20	67%
Watching CPR and the conduct of the participants	10	33%
Theme 4- Experience and reaction of the relative witnessing(or not) the resuscitation	26	87%
Family members presents:		
Feeling of relief in relation to the patient’s distress	7	23%
Experience of excessively heroic treatment and intrusion of shocking images	7	23%
Family members not presents:		
Experience of violence, brutality and dehumanization	5	17%

**Table 3:** Selected quotes.

**1. Choosing to be actively involved in the resuscitation**
*To be actively involved in the resuscitation process*
“I was present because I had already been participating [in the cardiac massage] from the beginning, when they arrived …As I had participated from the outset, I was in the main room, the living room, where my husband was, so … I think that it was too late in this case because I’d started participating at…so that’s it (relative #119)”
*To feel emotionally able to be present*
“I’m, umm, how do you say, not very emotional, so I would have liked to be there at the intervention … Because I said to him [to the emergency worker] that I felt ready and that I knew what to expect. For my part, because of my occupation, I can keep calm, perhaps a little better than average (relative #431)”
(…) “there’s nothing to worry about, if I don’t feel I can handle it, I’ll go out, but I’m staying to the end (relative #350)
*To support the patient during CPR*
“…be there, yes! Be present or hold his hand, or, I don’t know. I [emotion in her voice] held his hand to reassure him (…) (relative #71)
“I certainly want to be there to say, I’m here, next to you, I’m here for you …(relative #378)”
“(…) I think that the spirit is always there and that … perhaps he would not have liked me to turn away from him…(relative #499)
“I didn’t want her to stay alone (relative #431)”
*See the efforts of the resuscitation team*
“…I saw that they had done the maximum and that unfortunately, there wasn’t much they could do (relative #398)”
“…I saw, they really did what they could (relative #288)”
*Wish to protect oneself*
“No not at all (…) Because already the fact that how …how it happened, how he came out of the room to tell me to call an ambulance, how he collapsed and had spasms, I don’t know what he had. Already that, that haunts me, it’s in my head, and I don’t want to put more images like these (relative #192)”
**2. Communication between the family member and the emergency team**
*Medical information for the relative*
“They spoke to me from time to time … That’s it. They didn’t forget that I was there, let’s say (relative #288)”
“I found that they explained very well (relative #298)”
“They came from time to time to tell us what was going on. At one point, the woman said that his condition was very, very serious (relative #192)”
“it was important that they explained to us what was happening at that moment (relative #431)”
*Satisfaction (or dissatisfaction) about the medical team’s intervention*
“…they did the maximum (relative #378)”
“the whole team, the whole team, they have done their job, the whole team…and he [the doctor] he said…he was running around, and the whole team, they have done sufficiently (relative #427)”
“They were perfect, there was nothing to declare (relative #119)”
“They were vey good (. . .) very sweet, very nice, very attentive (…) (relative #194)”
“…conflict no (hesitation), disagreement and …how can I say it, incomprehension, especially about the organization and how it works, not disagreement with the people (relative #472)”
**3. Perception of the reality of death**
*Awareness of death at the arrival of the emergency team*
“but when they arrived, I knew that my wife was dead (relative #194)”
“yes, it’s true that, well, in any case it was (…) there was nothing to do. Well, it’s, I mean that it was very sudden for my father (relative #194)”
*Watching CPR and the conduct of the participants*
“Yes, yes and then this is what I want to tell you there, it was the exchange of looks between the person with the defibrillator and then the doctor who … so he was intubated and then finally, the whole thing, the bottles, everything, ventilated, um … and I said to myself: ‘They're not saying it, they're looking, they understand, and there's something, I can feel it's hard, it's serious!’ That what I felt (…) it's what I explained to friends, I felt them, the exchanges of looks between the emergency team, and then he was shocked, he wasn't reacting either, so I saw them look at each other, I said to myself: “Something's wrong! And then, well he started again, but good, uh. . . … (sigh) for me it was already over, I said to myself: ‘It's screwed’ (relative #362)”.
“because it was already 10 minutes that they’d been trying to resuscitate him, from when they arrived, at the end of 10 or 15 minutes, I asked if the heartbeat had resumed. They said no. It’s then that I started to understand that … well, that it was over! (relative #288)
**4. Experience and reaction of the relative witnessing (ot not) the resuscitation**
*Presence*: *Feeling of relief in relation to the patient’s distress*
“… if it was to resuscitate a vegetable, I prefer being in our situation today, even though I’m a widow, than to go to see my husband at the hospital as a vegetable (relative #519)”
*Presence*: *Experience of excessively heroic treatment and intrusion of shocking images*
“So he was lying on the ground, on a strange contraption [mechanical chest compression unit], he was … this image remains in my mind for few seconds (relative #430)”
*Absence*: *Experience of violence*, *brutality and deshumanization*
“We had the impression that he was an object rather than a human being (relative #353)
“We couldn’t know what was happening, how it happened and why it happened (relative #472)”

### Theme 1: choosing to be actively involved in the resuscitation

Family members often mentioned reasons to explain their willingness—or unwillingness—to be present during CPR.

#### To be actively involved in the resuscitation process

Participants frequently used the notion of being active in the resuscitation procedure to describe how they experienced their presence then. Some thought that their presence might have facilitated the resuscitation. The relatives interviewed expressed this in different ways. Some had begun cardiac massage before any emergency personnel arrived and stated that they felt they had been part of the process from the outset.

Others imagined that giving medical information to the team could provide missing information that would optimize the care: “*I'm the one who knows all of my mother's diseases*, *the doses of the drugs she takes*, *and everything…(relative #298)”*

#### To feel emotionally able to be present

When relatives agreed to be present, they frequently mentioned emotional reasons. Some linked their willingness to their personal previous experience of supporting relatives or friends: “*given that I was there all the way to the end for my grandfather*, *I wanted to be there [for my grandmother] (relative #431)”;* others attributed their willingness to be present to an innate character trait, in anticipating the emotional upheaval due to the situation. Others relatives knew they could cope even if they lost emotional control.

#### To support the patient during CPR

Support of the patient during the resuscitation procedures seems to be very important and is often mentioned as relief for the relative but also for the patient. It can include the need to: (1) touch the loved one; (2) provide moral support; (3) to establish a link beyond death with the loved one, by a spiritual intermediary; (4) maintain a form of communication with the patient “*be able to talk to the person*, *the person who died*, *who is in the process of dying (relative #473)”*.

#### To see the efforts of the resuscitation team

A direct understanding and appreciation of the resuscitation process appears determinant in helping family members to understand and accept the patient's death. To be certain that everything was tried to return the loved one to life provides, some relatives said, pacification. Having been a witness makes it possible to start to process the loss: “*And I think that it's important*, *it's part of the work of grieving also*, *to see that everything was tried and to truly see it oneself*, *I think that's very important (relative #547)*.*”*

Under the same main theme, we identified sub-themes about not being present during CPR:

#### Wish to protect oneself

Some family members reported wanting to protect themselves, especially from the CPR and images they judged disturbing linked to viewing some procedures. The possibly traumatic nature of these procedures, the need not to see the body assaulted by infusions, catheters, sensors, or simply the fear of experiencing a psychologically traumatizing event characterized this sub-theme: *“(…) I prefer to let them do it (…) I know*, *finally I understand it's sometimes procedures that are a little*…*uh…finally*, *uh…there are the*…*uh… finally that's it…(relative #421)”*. Some expressed especially the fear of adding other traumatic images to those of the collapse. None of included relatives had any previous experience with such medical procedures.

### Theme 2—Communication between the family member and the emergency team

The quality of the communication between the professionals and the family members was another theme mentioned very frequently in these relatives’ experience. Several sub-themes were identified.

#### Medical information for the relative

The feeling of being sufficiently well informed of the medical course of the management is mentioned by the relatives who did and did not witness the CPR: “*they explained everything to me all the way through (…) everything that was done during the intervention was explained to me…(relative #431)”*. The team’s consideration of the relative was experienced positively. The clarity of the explanations was often mentioned as a positive element: “*they were very clear*. *You'd have had to be stupid to not understand (relative #421)”*. Some relatives assessed the importance of this medical explanation and considered that the time devoted to explaining the severity of the patient's clinical state was sufficient.

#### Satisfaction (or dissatisfaction) with the medical team's intervention

This topic was most often associated with a feeling of satisfaction: *“…yes*, *it's true*! *I found they did their job really well (relative #288)”*. The doctor's actions in offering to allow the family to be present during CPR was most often experienced as a good thing: *“It's a good thing that they offer [to let you be present](…)(relative #473)”*. Some relatives expressed their satisfaction of having been treated appropriately by the team throughout the resuscitation. The dissatisfactions reported concerned an experience of lack of understanding and of disorganization in the intervention, revealed by lack of communication and interaction with the team: *“(…)I didn’t have any communication with the paramedics(…)the paramedics*, *they seemed to be robots at the orders of the ambulance doctor*, *and we didn't hear them at all (relative #073)”*.

### Theme 3- Perception of the reality of death

Seeing the body, observing the professionals’ efforts to bring the patient back to life and verbal communication with them, and viewing the nonverbal exchanges between the participants helped the family to understand the sudden death of their loved one

#### Awareness of death at the arrival of the emergency team

Family members mentioned the arrival of the emergency care team as a trigger for understanding the reality of the death and thus of the futility of resuscitation. The team's arrival confirmed the family's initial perception of severity, on viewing the collapse. “*Er*, *so I called the SAMU*, *um I gave them three very specific indications about the place and the circumstances; they asked me to check his condition*, *um*, *if he was really*…*um in a coma or*…*So I checked his condition and he was already*, *he was already blue in the face (relative #363)”*.

#### Watching CPR and the conduct of the participants

Directly watching these resuscitation procedures certainly plays a role in the genesis of the understanding that the person is dead. The duration of CPR, the rescue workers' failure to make the heart start to beat again—these factors facilitate the process of understanding the fatal outcome. This understanding can be facilitated by verbal communication between the participants, by communication that takes place progressively, spread out over the time of the intervention: “…t*hey came from time to time to tell us what was going on*. *At one point*, *the woman said that his condition was very*, *very serious…Then from that I said*, *‘well…I think that in my opinion there's not really anything left to do’ (relative #192)”*.

Nonverbal communication, especially how participants look at each other, was also mentioned as an element promoting awareness of this death.

### Theme 4- Experience and reaction of the relative witnessing (or not) the resuscitation

Witnessing the gestures of resuscitation aroused many emotional reactions. The anticipated fear of negative or positive reactions was often mentioned as a reason for their choice about being present for the resuscitation.

#### Presence: feeling of relief in relation to the patient's distress

The theme of the family's relief that the patient would have no sequelae due to the difficulty. Some family members stressed their observation that the patient did not suffer: *“during all the time they were treating him*, *he was in a coma the whole time*, *and he didn't suffer*, *not at all (relative #427)”*.

#### Presence: experience of excessively heroic treatment and intrusion of shocking images

Some family members retained negative images and a negative memory of viewing the CPR. The theme of aggressive overtreatment returned frequently: *“…but okay*, *when I saw that they were being so aggressive*, *I felt it as an aggression*, *an assault on my husband (relative #519)”*. Similarly, the intrusion of aggressive images linked to the technical aspects of CPR returned as negative elements: *“…finally there was a machine that performed a cardiac massage and that's an image that I have trouble getting rid of…(relative #519)”*.

#### Absence: experience of violence, brutality and dehumanization

When the family members were not asked to be present for the resuscitation manoeuvres, they reported auditory experiences associated with a strong feeling of exclusion: *“…to hear the pumps and everything*, *it was…ugh…But ok*, *it was very*, *very forceful to hear…(relative #192)”*. The feeling of exclusion was described as the inability to say goodbye: *“I couldn't say goodbye*, *as I wanted…(relative #071)*.

## Discussion

This ancillary qualitative study sheds light on how family members experience the patient’s resuscitation. We chose to use qualitative methods because they are increasingly recommended for exploring the results of randomized trials and identifying new avenues of research.[15–19]

Some of the themes and sub-themes found in our qualitative study have previously been evoked in the literature in observational or qualitative work, at least in part.[7,8,20,21] In a qualitative study of 14 interviews of parents present at their children’s resuscitations, the following themes were identified: those related to the perception of the reality of the resuscitation and to two needs: to be present near the child and to have a good relationship with the health care team.[8] The literature on the work of grieving has found a positive effect from offering family the choice of seeing the body of the loved one after death from cardiac arrest.[1,22] In a qualitative article assessing the feelings of family members after they saw the body of a relative who died traumatically, the authors found the themes of supporting the patient, of being able to say a last goodbye, and of the need to touch the body; they concluded that the ability to see the body was very positive, even one mutilated by a violent death.[23]

These themes were found in our work, as justifications of the need to be present (themes #1 and #3). Studying the cognitive mechanisms that might explain the beneficial effects of the family’s presence, Timmerman mentioned: 1) the need to soften the brutally sudden character of the events of the sudden death by communication with the medical team during the resuscitation, 2) the understanding that everything possible was done to save the person's life, 3) the observation that the patient was treated with respect and dignity, 4) facilitation of the process of the transition from life towards death.[24]

Other studies have shown that the quality of communication is one of the most important aspects for families.[25] This communication includes simultaneously verbal information but also empathy and support from these teams. Here the families stressed the simultaneous importance of verbal and nonverbal communication. Moreover, communication between health care personnel is directly affected by the family's presence.[21,26] A randomized trial tested a strategy of proactive communication towards the families of patients who died during resuscitation. The authors showed the beneficial effect of this strategy on the psychological consequences on the relatives by a significant reduction in the frequency of post-traumatic stress syndrome.[25]

Theme #4 suggests that the experience of watching resuscitation may be traumatizing. Some authors use this argument to deny families the choice to be present.[27,28] In a survey of 592 health-care professionals, 79% cited this point as the primary reason for not allowing families to be present at the resuscitation.[28] A trial based on this observation showed an increase in the frequency of PTSD in 34 families that were present for failed resuscitations.[11] Analysis of theme #4 indicates ambivalence between the potentially traumatizing nature of the technically sophisticated resuscitation, which could be experienced as aggressive overtreatment, and the need to be present to say goodbye. This ambivalence underlines the need to prepare and support the family and maybe even to see them again after the death.

What this study shows is the benefit of feeling oneself an active participant, of being able to choose whether or not to be present, and of being able to show self-efficacy in so complex and emotionally delicate a situation. This result fits into the concept of *sense of agency*,[29] defined as a person’s capacity to perceive him- or herself as able to master and control his or her environment [30], in contrast to passively undergoing a situation, being subjected to it. Offering family members the choice to be present during CPR contributes to developing this sense of agency. In addition, our results suggest that their presence has a positive effect on all 3 of its components: (i) perception (understanding of the reality of the death), (ii) cognition (thoughts related to support, and direct communication with the team), and (iii) emotions (experience of shock and relief). The health care team's consideration of the patient may facilitate an experience of active involvement in the resuscitation process. The feeling of active involvement counteracts the experience of helplessness and may thus be a factor that protects against traumatic grieving.

Our study is subject to limitations. First, the transferability of our findings should take into account the country-specific medical system. A second limitation, our conclusions should be generalized only with caution to different populations, including, but not limited to pediatric populations.

## Conclusion

Our results suggest that the practice of offering family members the choice of whether or not to view resuscitation has an emotionally protective effect in the face of this potentially traumatic event and thus call for the reconsideration of standard practices about CPR of patients in the presence of their immediate family members.

## Supporting Information

S1 Appendix Guide for semi-directive interview. (DOCX)
