# Supplementary material for: Family Presence during Resuscitation: A Qualitative Analysis from a National Multicenter Randomized Clinical Trial
Source: PLoS One. 2016 Jun 2;11(6):e0156100. doi: 10.1371/journal.pone.0156100 (PMC4890739; doi:10.1371/journal.pone.0156100)
Supplement: S1 Appendix — (DOCX) [file pone.0156100.s001.docx]

**S1 Appendix . Guide for semi-directive interview**

| **Questions and prompts** |
| --- |
| 1. Can you tell me how the emergency intervention took place? |
| How did you feel? |
| What was your state of mind? |
| What did you do? |
| What did you think about? |
| 2. Can you describe the interaction between you and the healthcare team? What were the steps related to the process of exchanging information? |
| How did you feel? |
| What did they do? |
| What did you do? |
| What did you think about? |
| 3. How did you feel having witnessed/ not witnessed the CPR? |
| Why? |
| Possible prompts: Can you tell more about that? Can you recall a particular example of that? |
